# Association of Menstrual Cycle with Fronto-Striatal Connectivity and Delay Discounting

**DOI:** 10.3390/bs15121747

**Published:** 2025-12-18

**Authors:** Ming Yang, Jiajia Xie, Xiaofen An, Jinying Zhuang

**Affiliations:** 1 School of Psychology and Cognitive Science, East China Normal University, Shanghai 200062, China; 52213200013@stu.ecnu.edu.cn (M.Y.);; 2 Department of Psychology, School of Education Science, Qingdao University, Qingdao 266071, China

**Keywords:** menstrual cycle, dorsolateral prefrontal cortex, caudate, functional connectivity, progesterone

## Abstract

The dorsal fronto-striatal circuit, particularly the pathway connecting the dorsolateral prefrontal cortex (dlPFC) and caudate, constitutes a core neural system for cognitive control and goal-directed behavior. While ovarian hormone fluctuations are known to influence this circuit, their precise impact on its role in decision-making remains poorly understood. Here, we leveraged the natural hormonal variation in the menstrual cycle to investigate how estradiol and progesterone shape dlPFC-caudate functional connectivity during a delay-discounting task. We discovered a state-dependent reconfiguration, characterized by the emergence of more negative connectivity for delayed rewards (vs. immediate rewards) in the mid-luteal phase and the dissipation of this pattern in the late follicular phase. Crucially, progesterone levels in the mid-luteal phase fine-tuned the circuit’s behavioral relevance, altering the association between connectivity strength and individual discounting rates. Our findings demonstrate that naturally occurring hormonal fluctuations reversibly reconfigure the functional architecture of the dorsal fronto-striatal circuit, thereby orchestrating state-dependent shifts in human decision-making.

## 1. Introduction

Human behavior is believed to emerge from parallel decision systems: a valuation system that estimates the value of different options and a control system that maintains future goals and inhibits prepotent responses (Van den Bos & McClure, 2012). By balancing these systems, individuals can make adaptive choices in complex environments. Conversely, dysregulation within either system can lead to maladaptive behaviors such as apathy, impulsivity, and addiction (Levy & Dubois, 2005; Passamonti et al., 2018; Wang et al., 2018).

The fronto-striatal loops are key neural substrates for these decisional processes (Van den Bos et al., 2014, 2015). Anatomically, these circuits comprise neural pathways connecting the frontal cortex and striatum, facilitating signal transmission between these regions (Averbeck & O’Doherty, 2021). Functionally, the ventral circuit—linking the ventral prefrontal cortex and ventral striatum—is implicated in representing option values, whereas the dorsal circuit, connecting the dorsal prefrontal cortex and dorsal striatum (DS), is critical for inhibiting prepotent responses (Peters & Büchel, 2011; Van den Bos et al., 2015).

Within the dorsal circuit, the dorsolateral prefrontal cortex (dlPFC) and caudate are central to goal-directed behavior and cognitive control (Bang et al., 2020; Cools, 2016; Turner et al., 2018). This inhibitory function is pivotal in intertemporal choice, where selecting a delayed reward requires suppressing the automatic preference for an immediate alternative. The delay-discounting task effectively probes this specific form of motivational impulse suppression, which is fundamental to real-world self-control (Bickel et al., 2011). Accordingly, dlPFC–caudate functional connectivity becomes more negative (suggestive of increased inhibitory signaling) when individuals choose delayed over immediate rewards, which is associated with greater patience (Van den Bos et al., 2014, 2015). Thus, examining this circuit during intertemporal choice allows direct investigation of how cognitive control overrides value-driven, prepotent responses.

Alterations in the dlPFC–caudate circuit have been linked to maladaptive behaviors. Its maturation correlates with reduced impulsivity in adolescents (Van den Bos et al., 2015), whereas circuit damage is associated with increased impulsivity and addiction (Yuan et al., 2016, 2017). Such findings suggest that shifts in behavioral preferences may stem from dorsal circuit dysfunction. For example, patients with anorexia nervosa exhibit more positive dlPFC–DS connectivity when selecting low-fat versus high-fat foods—a pattern reversed in healthy individuals (Foerde et al., 2015).

While extensive research has focused on pathological states, the mechanisms underlying normal physiological fluctuations in dlPFC–caudate circuitry remain poorly understood. The female menstrual cycle offers a natural model of such physiological variation. Accumulating evidence indicates that progesterone and estradiol—key ovarian hormones that fluctuate across the cycle—modulate activity in the dlPFC and caudate. Progesterone, which peaks in the mid-luteal phase (mid-LP), influences prefrontal dopaminergic transmission via its metabolite allopregnanolone (Motzo et al., 1996). Furthermore, allopregnanolone acts as a potent positive allosteric modulator of GABA-A receptors, enhancing inhibitory tone and potentially reconfiguring network states within the dlPFC (Guille et al., 2008; Barth et al., 2015). In contrast, estradiol, which peaks in the late follicular phase (late-FP), can mitigate NMDA receptor antagonist-induced cognitive deficits (Gogos et al., 2012) and finely coordinate dopaminergic transmission in the striatum (Levesque & Di Paolo, 1988; Schultz et al., 2009; Xiao & Becker, 1998). Consistent with these mechanisms, dlPFC activity during reward processing correlates positively with estrogen levels, and striatal activation is more positive in the follicular phase, suggesting heightened reward sensitivity (Dreher et al., 2007). Furthermore, females in the follicular phase show greater caudate activation during delay discounting, while those in the luteal phase exhibit enhanced dlPFC activation at rest, correlating positively with estradiol levels (Zhuang et al., 2020).

However, this evidence is not without controversy. As highlighted by Toffoletto et al. (2014), hormonal effects on brain function are diffuse and lack a consistent pattern. Inconsistencies extend to the behavioral level, where methodologically sound studies often fail to demonstrate robust menstrual cycle effects on cognitive performance (Le et al., 2020; Sundström-Poromaa, 2018; Sundström-Poromaa & Gingnell, 2014). For instance, while some studies report cycle-dependent alterations in dlPFC–caudate circuits (e.g., Zhuang et al., 2020), others find no significant menstrual cycle effects on resting-state networks in larger meta-analyses (Hjelmervik et al., 2014). A significant limitation in the existing literature is the reliance on a narrow set of experimental paradigms, which may not fully capture the nuanced effects of ovarian hormones on goal-directed behavior and cognitive control (Ambrase et al., 2021). Consequently, direct evidence linking hormonal fluctuations to dynamic changes in dlPFC–caudate connectivity and decision-making remains scarce.

To address this gap, the delay-discounting task offers a powerful alternative. It is a well-validated measure of impulse suppression central to real-world self-control and is sensitive to behavioral changes across clinical populations (Bickel et al., 2019; Chen et al., 2021). Critically, its neural basis is well-established in the dorsal fronto-striatal circuit, where dlPFC-caudate connectivity is essential for implementing patience and overriding prepotent responses for immediate reward (Van den Bos et al., 2014, 2015). Moreover, dynamic connectivity patterns in this circuit during intertemporal choice have been shown to predict real-world behaviors such as caloric intake (Foerde et al., 2015). Nevertheless, direct evidence linking hormonal fluctuations to these dynamic changes in dlPFC-caudate circuitry during delay discounting is still lacking.

Therefore, to investigate how natural hormonal fluctuations modulate the dorsal circuit’s role in suppressing value-driven impulses, we combined functional magnetic resonance imaging (fMRI) with a delay-discounting task across different menstrual cycle phases. We hypothesized that dlPFC–caudate functional connectivity would differ significantly depending on participants’ choices, and that menstrual cycle phase would modulate the association between this connectivity and delay discounting through fluctuations in ovarian hormones.

## 2. Materials and Methods

### 2.1. Participants

We recruited 24 female students (mean age, 20.72 years; standard deviation (SD), 2.05 years) from the university community to participate in this study. This sample size was established following a relevant fMRI-based study conducted by Zhuang et al. (2020). All participants were healthy and right-handed, with normal or corrected-to-normal vision, no reported medication use, and no history of psychological or neurological conditions. They had no history of hormonal contraceptive use in the past 6 months and regular (28–30-day) menstrual cycles. For each participant, we used forward counting from the cycle onset day (day 1) to predict the late FP (days 12–14) and mid-LP (days 20–22; Wilcox et al., 2000).

The Ethics Committee of East China Normal University approved the study protocol. All participants provided written informed consent before the experiment and received 100 RMB as payment after they had completed the study.

### 2.2. Salivary Samples

Saliva samples were collected via passive drool into sterile tubes immediately upon participants’ arrival at the laboratory, following a 1 h fast and 30 min fluid abstinence. Approximately 2 mL of saliva was obtained from each participant. All samples were immediately frozen and stored at −80 °C until batch analysis. Salivary concentrations of estradiol and progesterone were quantified using commercial enzyme-linked immunosorbent assay (ELISA) kits (Estradiol: Catalog Number SLVV-4188; Progesterone: Catalog Number SLV-2931; DRG International, Inc., Springfield, NJ, USA). The assays’ lower limits of detection (LLOD) were 0.4 pg/mL for estradiol and 3.9 pg/mL for progesterone. According to the manufacturer’s specifications, the intra-assay coefficients of variation (CVs) ranged from 2.1% to 3.8% for estradiol and from 5.3% to 7.7% for progesterone. The inter-assay CVs ranged from 2.6% to 6.9% for estradiol and from 4.7% to 7.6% for progesterone. The observed inter-assay CVs in our study were 6.47% for estradiol and 13.40% for progesterone, which fall within the acceptable range reported in the literature for salivary hormone assays (Herrera et al., 2016).

### 2.3. Task

We employed a within-subjects (repeated-measures) design to investigate the effects of menstrual cycle phase. Each participant performed a delay-discounting task in the fMRI scanner during the late FP and mid-LP. The task was the same in both cases. Sixteen of the participants were examined first in the late FP. The testing order was included as a covariate in subsequent statistical analyses to account for its potential influence.

**Figure 1:**
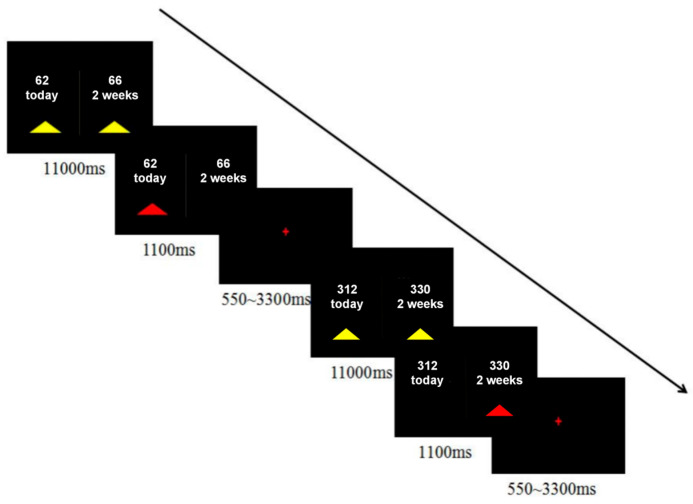
Experimental design. The task consisted of 40 binary hypothetical choices, the order of which was randomized among participants. Decisions were self-paced, with a maximum allowed reaction time of 11,000 ms. A feedback screen indicating the choice outcome was presented for 1100 ms after each response.

For the delay-discounting task, each participant made 40 binary, hypothetical choices between a smaller-sooner (SS) and a larger-later (LL) reward (Figure 1). The SS delays were “Today”, while the LL delays were “2 weeks”, “4 weeks”, or “6 weeks”. Reward amounts were adjusted so that the percentage difference, (LL − SS)/SS, varied across eight levels (1%, 3%, 5%, 10%, 15%, 25%, 35%, and 50%). The specific amounts in Renminbi (RMB), ranging from approximately ¥31.25 to ¥350, were derived by converting the original US dollar values from Kim et al. (2012) and halving them. Participants indicated their preference by pressing one of two buttons with their right hand as soon as the choice appeared. Decisions were self-paced, with a maximum allowed reaction time of 11,000 ms. A feedback screen displaying the chosen outcome was shown for 1100 ms, followed by a jittered inter-trial interval of 550–3300 ms. The entire task lasted approximately 7 min and was presented using the in vivo Esys system for fMRI (Gainesville, FL, USA).

### 2.4. Imaging Data Acquisition

We recorded the time of day for each scanning session (scanning time) to account for potential diurnal confounds. All sessions occurred between 13:00 and 19:00, with the specific time documented for subsequent sensitivity analyses (see Supplementary Material). MR images were acquired using a 3.0-Tesla Siemens (Erlangen, Germany) Trio Tim scanner with a 32-channel head coil at the fMRI laboratory of East China Normal University. T1-weighted sagittal structural images were acquired with the following parameters: repetition time (TR) = 2530 ms, echo time (TE) = 2.34 ms, field of view (FOV) = 256 mm, flip angle = 7°, voxel size = 1 × 1 × 1 mm, and 192 slices covering the whole brain. Functional images were obtained using a gradient echo-planar imaging sequence (TR = 2200 ms, TE = 30 ms, FOV = 220 mm, flip angle = 81°, voxel size = 3.44 × 3.44 × 3.9 mm, 35 slices oriented parallel to the anterior and posterior commissure covering the whole brain).

### 2.5. Behavioral Data Analysis

We employed a calculation method similar to that used by Zhuang et al. (2020), fitting the behavioral data using a hyperbolic discount function:

*V*(*r*,*t*) = *r*/(1 + *kt*), (1)

where *r* is the reward amount available at delay *t*, *V* is the subjective value of the offer, and *k* is the discount rate, estimated for each subject using a logistic decision function with the maximization of the log-likelihood of the observed choices. The best-fitting model parameters were determined using Matlab R2019b (the MathWorks, Natick, MA, USA) via toolbox (Lau, 2016). The hyperbolic discounting parameter *k* was estimated separately for each menstrual cycle phase (late FP and mid-LP) for each participant, yielding two independent discount rates per subject: *k*_FP and *k*_LP. To evaluate the effect of menstrual cycle phase while controlling for the imbalanced testing order, we fitted a linear mixed-effects model to the log-transformed discount rates (log *k*). The model included fixed effects for phase (late FP vs. mid-LP), testing order and scanning time, with a random intercept for subject. For direct comparison, a paired-samples *t*-test was also performed on the log *k* values.

### 2.6. fMRI Data Processing

The fMRI data were preprocessed and analyzed using Statistical Parametric Mapping (SPM) software (version 8; The Wellcome Department of Imaging Neuroscience, London, UK). We first performed slice-timing correction, then realigned the functional images to the first image to correct for head movements. Images from participants with excessive head movement (>3 mm translation and >3° rotation) were excluded. Next, each T1-weighted three-dimensional structural image was co-registered to the mean echo planar image generated after realignment, then segmented into gray matter, white matter, and cerebrospinal fluid components using a unified segmentation algorithm. After realignment, the functional images were normalized to Montreal Neurological Institute (MNI) space (resampled to 2 × 2 × 2 mm^3^) using the normalization parameters estimated during unified segmentation, and then spatially smoothed with an 8 mm full-width at half-maximum Gaussian kernel.

To ensure that our functional connectivity results were not confounded by head motion, we conducted supplementary motion-sensitivity analyses. The procedures and results are detailed in Supplementary Material S1 and confirm no significant relationship between motion and our key finding.

### 2.7. Whole-Brain Analysis

Parametric mapping of each subject’s local brain activation was performed using a standard generalized linear model. For the first level of the analysis, we used an event-related design to estimate neural responses to events of interest. Potentially confounding variables, such as trial-by-trial head movements and choice outcomes (i.e., motor responses), were included in this model as regressors of no interest. We pooled all r1 choices to form the immediate reward choice condition and all r2 choices to form the delayed choice condition. Each overall condition [immediate reward choice in the late FP (FI), immediate reward choice in the mid-LP (LI), delayed reward choice in the late FP (FD), and delayed reward choice in the mid-LP (LD)] was modeled using the reaction times from decision onset and convolved with a canonical hemodynamic response function and its time derivatives. The six movement parameters were included as covariates of no interest. High-pass temporal filtering with a cut-off of 128 s was applied to remove low-frequency signal components. Simple main effects were computed for each participant and condition by applying appropriate t-contrasts (e.g., [1 0]) to estimate activity for each condition relative to baseline.

To identify brain regions related to the four conditions, we conducted a group-level analysis with a random effects model [flexible factorial analysis of variance (ANOVA)] using the SPM software. The effect between the menstrual phase and delay discounting was calculated using the contrast [(LI − LD) − (FI − FD)]. These analyses were performed for the whole brain using a cluster-level threshold of *p* < 0.05 (familywise error–corrected) and a voxel-level threshold of *p* < 0.001 (uncorrected). Activations were identified using the Anatomical Automated Labeling atlas (Tzourio-Mazoyer et al., 2002) in MNI space.

Statistical significance for whole-brain analyses was assessed using cluster-based inference. A cluster-forming threshold of *p* < 0.001 (uncorrected) at the voxel level was first applied. Contiguous voxels surviving this threshold were defined as clusters, and the significance of each cluster’s spatial extent was then assessed at the cluster level, with a family-wise error (FWE) correction threshold of *p* < 0.05. This procedure was implemented using SPM8’s Gaussian Random Field theory.

### 2.8. Functional Connectivity Analysis

To test our neural circuitry hypothesis, we conducted beta-series correlation (BSC) analyses of the functional connectivity between the caudate and dlPFC under each condition (FI, LI, FD, and LD). We defined regions of interest (ROIs) based on a priori coordinates derived from independent literature and modeled each trial as a separate event of interest (Rissman et al., 2004). The dlPFC coordinate (MNI: [36, 46, 31]) was selected from study of cognitive control circuits (Yuan et al., 2016). The caudate coordinate (MNI: [6, 26, −4]) was taken from study on delay-discounting choice (Koban et al., 2023). The beta series associated with each trial type in each ROI was extracted, and these series were sorted by the study condition. Pair-wise BSC analyses were performed for the DS and dlPFC. The correlation between activities in each ROI pair for each subject and condition across the time series was calculated, and the correlation values were subjected to Fisher transformation prior to inclusion in ANOVAs performed to identify correlations that varied across delay-discounting choices and menstrual phases.

Functional connectivity was analyzed using weighted linear mixed-effects models. The primary model included fixed effects for menstrual cycle phase (late FP, mid-LP), choice type (immediate, delayed), and their interaction, while controlling for testing order and scanning time as covariates. A random intercept was specified for subjects to account for within-participant dependencies. To address the variability in the reliability of beta-series correlations, each data point (representing a participant’s connectivity estimate in a specific condition) was weighted by the square root of the number of trials contributing to that estimate. Parameters were estimated using maximum likelihood, and significance was assessed with Type III F-tests with Satterthwaite degrees of freedom approximation. For significant interactions, simple effects analyses (pairwise comparisons) were conducted within the same weighted modeling framework, applying family-wise error (FWE) correction via the Bonferroni method.

### 2.9. Moderated Regression Analysis of Hormonal Effects

Following Foerde et al. (2015), we conducted analyses between discount rate (transformed *k*) and dlPFC–caudate connectivity differences (during delayed vs. immediate choices). We pre-specified a primary moderation analysis testing the three-way interaction between menstrual cycle phase, dlPFC-caudate functional connectivity difference, and progesterone levels on delay discounting rates, while controlling for testing order and scanning time. Connectivity difference and progesterone values were converted to z-scores (mean-centered and scaled by standard deviation) to facilitate interpretation. To account for differences in the reliability of functional connectivity estimates due to varying trial distributions across choice conditions, we implemented a trial-count weighting scheme. Each observation was weighted by the square root of the harmonic mean of the trial counts in the immediate and delayed choice conditions. This approach gives greater importance to connectivity difference scores derived from more balanced trial distributions, which provide more reliable estimates of the true neural contrast. Given sample size constraints, we employed weighted bootstrap linear regression with 5000 resamples, using participant as a stratification variable. This approach provides robust inference that approximates cluster resampling while incorporating trial-count reliability weights. We applied Bonferroni correction to control the family-wise error rate across all pre-specified tests. Model diagnostics included checks for R^2^, residual patterns, and the number of bootstrap resamples in the weighted regression framework. Following the significant three-way interaction, we conducted post hoc analyses to characterize the nature of this interaction across menstrual cycle phases. We performed separate weighted bootstrap linear regression models within each phase to examine the two-way interaction between functional connectivity difference and progesterone levels. For these phase-specific analyses, we maintained the same trial-count weighting scheme and bootstrap procedures (5000 resamples with participant stratification).

## 3. Results

### 3.1. Salivary Hormone

Hormone concentrations for each menstrual phase group were reported in Table 1. A paired-sample *t*-test revealed significant higher progesterone levels in the mid-LP group than in the late FP group (*t*(23) = 2.82, *p* = 0.01, mean difference = 14.53, 95% CI [3.86, 25.20], Cohen’s *d* = 0.58); estrogen levels were similar between these two groups (*t*(23) = −1.28, *p* = 0.22, mean difference = −0.61, 95% CI [−1.61, 0.38], Cohen’s *d* = 0.26). To control for potential confounding effects of scanning time, we included this variable in subsequent sensitivity analyses (see Supplementary Material).

### 3.2. Behavioral Results

All task responses were submitted well before the 11,000 ms time limit. We analyzed reaction times (RTs) to examine potential influences of the menstrual cycle and choice type on decision speed. A 2 (phase: late FP, mid-LP) × 2 (choice type: immediate, delayed) repeated-measures ANOVA was conducted on the log-transformed RTs (see Table 2). The analysis revealed no significant main effect of phase [*F*(1, 23) = 0.86, *p* = 0.36, *n_p_*^2^ = 0.04], indicating that overall response speed did not differ between the late FP (median = 1884 ms) and the mid-LP (median = 1769 ms). There was also no significant main effect of choice type [*F*(1, 23) = 2.78, *p* = 0.11, *n_p_*^2^ = 0.11], with comparable RTs for immediate choices (median = 1750 ms) and delayed choices (median = 1903 ms). The phase × choice type interaction was also not significant [*F*(1, 23) = 0.24, *p* = 0.63, *n_p_*^2^ = 0.01].

**Figure 2:**
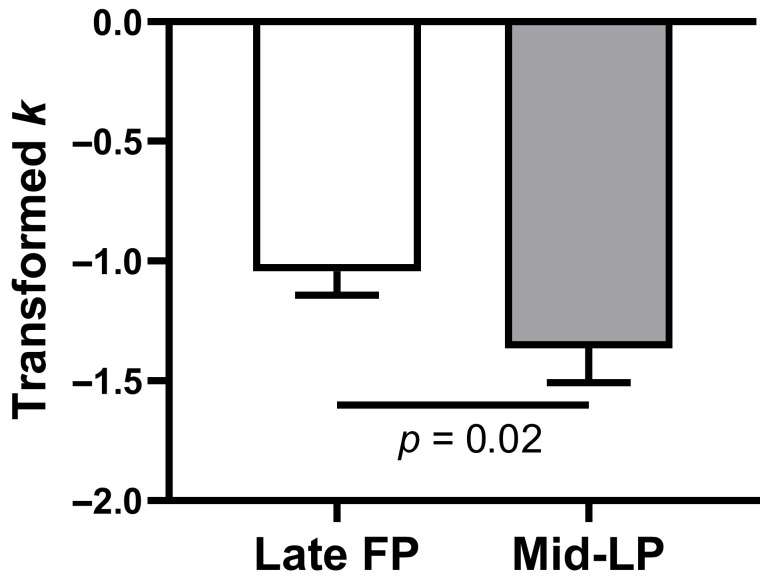
Transformed discount rates (log *k* values) by menstrual phase. Error bars indicate the standard error of the mean. FP, follicular phase; LP, luteal phase.

A linear mixed-effects model was used to analyze the log *k* with fixed effects for phase and order, and a random intercept for subject. This analysis revealed a significant main effect of phase (*β* = 0.60, SE = 0.21, *t*(43) = 3.52, *p* = 0.001, 95% CI = [0.26, 0.95]), with no significant effect of order (*β* = −0.27, SE = 0.21, *t*(43) = −1.30, *p* = 0.20, 95% CI = [−0.69, 0.15], Table 3). Consistent with this result, a paired-samples *t*-test confirmed that log(*k*) was significantly higher in the late FP than in the mid-LP (mean ± SE, −1.04 ± 0.10 vs. −1.36 ± 0.14; *t*(23) = 2.45, *p* = 0.02, mean difference = 0.32, 95% CI = [0.05, 0.59], Cohen’s *d* = 0.50; Figure 2).

### 3.3. Whole-Brain Findings

No participant had to be excluded due to excessive head movement during fMRI scanning. The whole-brain analysis for [(LI − LD) > (FI − FD)] showed stronger activation of the bilateral caudate (Table 2). The analysis of the reverse [(FI − FD) > (LI − LD)] showed activation in several areas [the dlPFC (in the bilateral superior frontal gyrus), left superior temporal gyrus, bilateral anterior cingulate cortex, right middle frontal gyrus, left angular gyrus, left middle temporal gyrus, right temporal pole, right thalamus, left inferior parietal lobule, right insula, right inferior temporal gyrus, and right cerebellum; Table 4].

### 3.4. Functional Connectivity

All functional connectivity analyses account for variability in estimation reliability due to differing trial counts across conditions and participants. The functional connectivity analysis was conducted using the a priori defined ROIs described in the Methods section (dlPFC: [36, 46, 31]; caudate: [6, 26, −4]).

**Figure 3:**
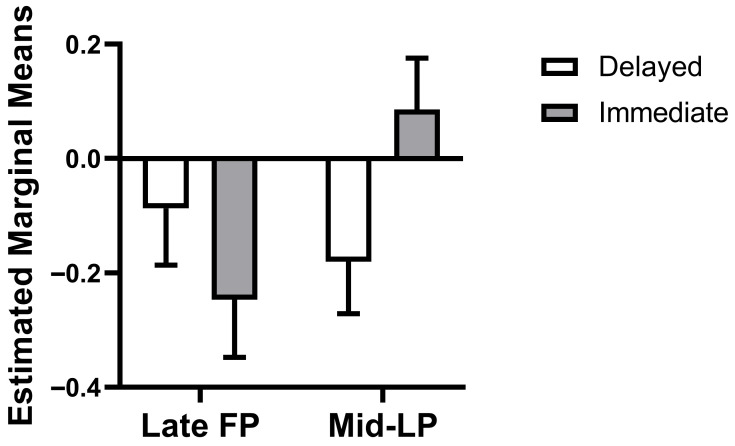
Phase × choice type interaction on dlPFC-Caudate connectivity. Bars represent estimated marginal means (±SEM). FP, follicular phase; LP, luteal phase.

The weighted mixed-effects model revealed a significant phase × choice type interaction (*β* = 0.42, SE = 0.17, *t*(70.12) = 2.49, *p* = 0.01, 95% CI = [0.09, 0.76]), while main effects of phase (*β* = −0.16, SE = 0.13, *t*(70.19) = −1.25, *p* = 0.22, 95% CI = [−0.41, 0.10]) and choice type (*β* = −0.09, SE = 0.12, *t*(72.01) = −0.77, *p* = 0.45, 95% CI = [−0.33, 0.15]) were not significant. This interaction reflects a qualitative reversal in dlPFC-caudate connectivity patterns across the menstrual cycle. Specifically, this interaction manifested as a reversal in the connectivity pattern: estimated marginal means showed that during the mid-LP, connectivity was more negative for delayed choices than for immediate choices (mean difference = −0.33, SE = 0.12, *df* = 71.41, *p* = 0.008, 95% CI = [−0.57, −0.09], Bonferroni-corrected α = 0.025). In the late FP, however, the pattern was numerically reversed and non-significant (mean difference = 0.09, SE = 0.12, *df* = 72.01, *p* = 0.45, 95% CI = [−0.15, 0.33], Figure 3).

To further quantify the evidence for this interaction, we performed a supplementary model comparison. A likelihood ratio test confirmed that the model containing the phase × choice interaction provided a significantly better fit to the data than a reduced model without the interaction term (χ^2^(1) = 6.10, *p* = 0.001).

Supplementary motion-sensitivity analyses confirmed that this interaction effect was not significantly correlated with head motion and did not differ between high- and low-motion participants (see Supplementary Material S1).

### 3.5. Moderating Effects of Hormone on Functional Connectivity and Discount Rates

To investigate how the phase-dependent connectivity patterns related to behavior, we fitted a linear model examining the relationship between functional connectivity and delay discounting rates. Our pre-specified primary analysis tested the three-way interaction between menstrual cycle phase, functional connectivity difference, and standardized progesterone levels, with observations weighted by trial-count reliability. The weighted bootstrap regression model (5000 resamples) revealed a significant three-way interaction (*β* = −1.61, bootstrap SE = 0.71, 95% CI [−2.70, −0.05], *p* = 0.011, Bonferroni-corrected *α* = 0.0125). The model also indicated two-way interactions between phase and progesterone (*β* = 0.96, bootstrap SE = 0.41, 95% CI [0.03, 1.73], *p* = 0.015) and between progesterone and functional connectivity (*β* = 1.08, bootstrap SE = 0.68, 95% CI [−0.53, 2.07], *p* = 0.05), though neither survived Bonferroni correction (*α* = 0.0125). Finally, the phase by functional connectivity interaction was not significant (*β* = 0.13, bootstrap SE = 0.40, 95% CI [−0.59, 1.02], *p* = 0.69).

**Figure 4:**
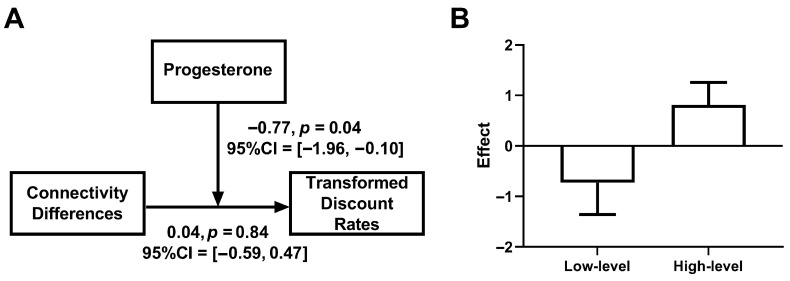
Moderating effects of progesterone on functional connectivity and discount rates during the mid-LP. (**A**) the moderating model revealed that progesterone concentrations moderated the relationship between dlPFC–caudate connectivity differences and discount rates. (**B**) the effects of connectivity differences on discount rates under high and low progesterone levels.

To characterize the three-way interaction, we conducted phase-specific analyses of the connectivity difference × progesterone moderation. During the mid-LP, progesterone significantly moderated the relationship between connectivity difference and discounting (*β* = −0.77, bootstrap SE = 0.46, 95% CI [−1.96, −0.10], *p* = 0.04, Figure 4A). Simple slope analyses revealed a qualitatively opposite pattern of associations across progesterone levels: at high progesterone levels (+1 SD), there was a trend toward a positive association between connectivity difference and discount rate (*β* = 0.81, bootstrap SE = 0.45, 95% CI [−0.32, 1.48], *p* = 0.06), and this relationship was numerically reversed at low progesterone levels (−1 SD; *β* = −0.73, bootstrap SE = 0.63, 95% CI [−2.50, 0.23], *p* = 0.08, Figure 4B). It should be noted that both simple effects were non-significant both before and after Bonferroni correction (*α* = 0.0125). In contrast, during the late FP, the connectivity difference × progesterone interaction was non-significant (*β* = 1.18, bootstrap SE = 1.16, 95% CI [−1.37, 3.16], *p* = 0.20).

## 4. Discussion

This study investigated how the menstrual cycle influences intertemporal choice and its neural circuitry. We found that delay discounting was steeper (i.e., choices were more impulsive) in the late FP compared to the mid-LP. At the circuit level, this was accompanied by a qualitative reversal in the functional coupling pattern of the dlPFC-caudate circuit during choice, which was significantly different between the late FP and mid-LP. Furthermore, progesterone levels during the mid-LP modulated how this circuit dynamic related to impulsive behavior. To ensure the robustness of our primary finding, we rigorously controlled for potential confounding factors, such as testing order and time of day. Critically, our findings remained highly significant after accounting for these variables. This confirms that the cyclical reversal in connectivity patterns is a robust phenomenon, independent of these potential confounds, and underscores the profound impact of menstrual cycle phase on this cognitive control circuit.

Our study establishes that the menstrual cycle dynamically reconfigures the functional architecture of the dorsal fronto-striatal circuit during intertemporal choice. We observed a qualitative reversal in dlPFC-caudate connectivity patterns: during the mid-LP, coupling was more negative for delayed versus immediate rewards, directly aligning with the canonical pattern associated with increased patience and successful self-control in healthy populations (Van den Bos et al., 2014, 2015). In striking contrast, this pattern was absent during the late FP. This state-dependent shift in circuit operation provides a potential neural substrate for documented cognitive fluctuations across the menstrual cycle (Li et al., 2022; Xie et al., 2021). For instance, the late FP has been associated with heightened reward sensitivity and greater caudate activity (Zhuang et al., 2020). Conversely, the mid-LP is frequently linked to enhanced cognitive control and increased dlPFC engagement (Hidalgo-Lopez & Pletzer, 2021), consistent with the lower discounting rates (greater patience) we observed in this phase.

This reconfiguration finds a parsimonious account in the Dual Mechanisms of Control (DMC) framework (Braver, 2012). The significant negative connectivity for delayed rewards observed in the mid-LP strongly reflects a proactive control mode, characterized by the sustained, anticipatory maintenance of goal representations to preemptively bias choice toward long-term outcomes. Conversely, the absence of a significant connectivity difference in the late FP is consistent with a greater reliance on a reactive control strategy, engaged transiently to resolve decision conflict. This interpretation aligns with the broader principle of dynamic network reconfiguration across brain states (Shine & Poldrack, 2018). The shift toward proactive control in the mid-LP is consistent with the documented role of progesterone in enhancing prefrontal function and cognitive control (Berent-Spillson et al., 2015; Song & Wang, 2025), potentially underpinning a protective mechanism against impulsive decision-making in females.

Critically, progesterone levels during the mid-LP served as a key modulator of this circuit-behavior relationship. At the higher progesterone levels, a more positive connectivity difference was associated with a trend toward a higher discount rate. This pattern converges with the established literature linking a less negative (or more positive) fronto-striatal coupling to more impulsive choice (Van den Bos et al., 2014, 2015). Strikingly, at lower progesterone levels, this trend was reversed. This suggests that progesterone does not simply enhance cognitive control in a linear fashion but modifies how the dorsal circuit implements control. This modulatory role is supported by evidence that progesterone promotes prefrontal-dependent functions and dampens reward-seeking (Berent-Spillson et al., 2015; Lynch & Sofuoglu, 2010). potentially through its influence on neural excitability and network dynamics (Song & Wang, 2025). We therefore posit that progesterone acts as a key physiological signal that biases the brain toward a proactive, controlled state, implementing a protective mechanism against impulsive choices (Lynch & Sofuoglu, 2010).

Several methodological considerations should guide future research. First, while our sample size provided sufficient power to detect the robust interaction effects central to our hypothesis, larger cohorts will enable the investigation of more nuanced hormone-behavior relationships. Second, our design and measures do not allow us to dissociate whether the observed neural reconfiguration primarily influences the computation of subjective value or the control of impulsive choice, a crucial distinction for future research to address. Third, our focus on young adults (19–25 years) aimed to investigate hormonal effects within a period of relative neurodevelopmental stability, reducing confounding variance from adolescent maturation or perimenopausal transitions. It remains an open question how these hormone-circuit interactions evolve across the lifespan.

Our study has several limitations. First, while our findings suggest the role of progesterone in the mid-LP, cognition during this phase likely involves complex interactions between progesterone and estradiol, both of which remain at higher levels (Hall, 2009; Bernal & Paolieri, 2022). These hormones interact intricately, producing antagonistic or synergistic effects across cognitive domains (Baudry et al., 2013; Gould et al., 1990). Consequently, recent studies have advocated for the inclusion of more menstrual cycle phases to better disentangle the effects of different hormones (Bernal & Paolieri, 2022). Second, without direct measurement of dopamine levels, our approach precluded us from examining a compelling alternative explanation: that hormonal fluctuations influence the dlPFC–caudate circuit via dopaminergic pathways. Existing evidence supports this mechanism: both dlPFC and caudate are densely innervated by dopaminergic neurons (Bromberg-Martin et al., 2010), and dopamine levels in these regions predict delay discounting (Smith et al., 2014; Wagner et al., 2020). Moreover, estrogen modulates striatal dopamine release and receptor expression (Schultz et al., 2009; Xiao & Becker, 1998; Auger et al., 2001), while progesterone affects prefrontal dopamine transmission (Motzo et al., 1996). Thus, the observed phase-dependent reversal in connectivity may reflect dopamine-mediated effects. Future work should incorporate direct neurochemical measures to test this hypothesis.

In conclusion, our study establishes that the functional architecture of the human dorsal fronto-striatal circuit is dynamically reconfigured by natural physiological states. The menstrual cycle orchestrates a qualitative reversal in dlPFC-caudate connectivity during decision-making, a shift that is finely calibrated by progesterone and potentially reflects a transition between proactive and reactive control modes. These findings reveal a fundamental plasticity in the neural circuits governing choice, demonstrating that core aspects of human decision-making are intrinsically linked to the rhythmic hormonal cycles that define the female physiology.

## Figures and Tables

**Table 1:** Hormone concentrations (Mean ± SE) for menstrual phase.

Hormone	Menstrual Phase	
	Late FP	Mid-LP
Estradiol, pg/mL	2.69 ± 0.60	2.08 ± 0.97
Progesterone, pg/mL	11.26 ± 1.12	25.79 ± 5.16

**Table 2:** Reaction time (RT) descriptive statistics and ANOVA results.

**Descriptive Statistics**			
**Phase**	**Choice Type**	**Median RT (ms)**	**IQR (ms)**
Late FP	Immediate	1664	[1247–2157]
Late FP	Delayed	1673	[1283–2541]
Mid-LP	Immediate	1621	[1328–1897]
Mid-LP	Delayed	1897	[1335–2295]
**Inferential Statistics (RM-ANOVA)**			
**Effect**	**F-Value**	***p*** **-Value**	**Effect Size (*n_p_*^2^)**
Main Effect: Phase	*F*(1, 23) = 0.86	0.36	0.04
Main Effect: Choice	*F*(1, 23) = 2.78	0.11	0.11
Phase × Choice Interaction	*F*(1, 23) = 0.24	0.63	0.01

**Table 3:** Comparison of delay discounting rates (log *k*) between menstrual cycle phases.

Measure	Late FP	Mid-LP
**Delay Discounting Rate (log *k*)**		
Mean ± SE	−1.04 ± 0.10	−1.36 ± 0.14
**Statistical Comparison**	Paired *t*-test: *t*(23) = 2.45, *p* = 0.02	
**Effect Size**	Cohen’s *d* = 0.50	
**Mean Difference [95% CI]**	0.32 [0.05, 0.59]	

**Table 4:** Whole-brain activation during delay-discounting task performance.

Cluster	*k*	Maximum Peak Region	BA	H	*t*	MNI Space		
						x	y	z
(LI − LD) > (FI − FD)								
1	510	Caudate	48	L	5.05	−10	22	4
(FI − FD) > (LI − LD)								
1	604	Superior temporal gyrus	13	L	5.27	−42	2	−10
2	430	Superior frontal gyrus	6	L	4.61	−22	26	60
3	203	Anterior cingulate cortex	24	L	4.73	−2	2	28
4	180	Middle frontal gyrus	8	R	4.12	40	30	46
5	120	Angular gyrus	39	L	4	−52	−66	30
6	95	Middle temporal gyrus	21	L	4.4	−48	−12	−22
7	78	Temporal pole	38	R	3.32	44	14	−18
8	69	Anterior cingulate cortex	32	R	3.87	8	32	14
9	51	Thalamus	50	R	4.92	16	−34	6
10	49	Inferior parietal lobule	7	L	3.8	−30	−76	50
11	44	Anterior cingulate cortex	32	L	3.98	−4	36	22
12	40	Superior frontal gyrus	6	R	4.02	16	26	54
13	40	Insula	13	R	3.37	32	24	0
14	24	Inferior temporal gyrus	41	R	3.63	52	−10	−26
15	23	Cerebellum		R	4.07	16	−50	−46

Results are reported for cluster sizes > 20 with significant results (*p* < 0.05) after familywise error correction for multiple comparisons. *k*, cluster size; BA, Brodmann area; H, hemisphere; MNI, Montreal Neurological Institute; L, left; R, right.

## Data Availability

The data and code that support the findings of this study are openly available in the Open Science Framework (OSF) at [https://osf.io/cw5y6/overview?view_only=2d251af9d3de4676a7914c4ae802731d] (accessed on 5 December 2025).

## Supplementary Materials

The following supporting information can be downloaded at: https://www.mdpi.com/article/10.3390/bs15121747/s1, Table S1: Scanning order and session times for all participants; Table S2: Number of valid trials per condition and participant; Table S3: Diagnostics for linear moderation models; Methods & Results S1: Sensitivity Analyses for Time-of-Day Effects; Methods & Results S2: Motion Sensitivity Analyses.
