# Supplementary material for: Association of Menstrual Cycle with Fronto-Striatal Connectivity and Delay Discounting
Source: Behav Sci (Basel). 2025 Dec 18;15(12):1747. doi: 10.3390/bs15121747 (PMC12729421; doi:10.3390/bs15121747)
Supplement: Supplementary file 1 [file behavsci-15-01747-s001.zip › behavsci-3914653-supplementary.pdf]

Supplementary Materials Contents

Item	Page
**Supplementary Tables**	
Table S1. Scanning order and session times for all participants	S2
Table S2. Number of valid trials per condition and participant	S3
Table S3. Diagnostics for linear moderation models	S4
**Supplementary Methods and Results**	
S1. Sensitivity Analyses for Time-of-Day Effects	S5
S2. Motion Sensitivity Analyses	S8

Supplementary Table S1. Participant scanning order and timing of scanning sessions.

Subject ID	Scanning order (first phase)*	Time of first scan	Time of second scan
1	late FP	18:55	18:51
2	late FP	19:08	17:50
3	late FP	14:41	12:03
4	late FP	19:14	14:14
5	late FP	18:33	15:13
6	mid-LP	19:05	16:35
7	late FP	16:26	15:37
8	late FP	13:58	17:52
9	late FP	18:34	17:44
10	mid-LP	16:39	19:30
11	late FP	19:16	18:25
12	mid-LP	15:32	13:37
13	mid-LP	18:36	19:24
14	late FP	18:55	18:45
15	mid-LP	17:55	18:57
16	late FP	12:42	16:18
17	mid-LP	17:16	18:17
18	late FP	19:05	15:56
19	late FP	11:22	19:15
20	mid-LP	18:31	17:19
21	late FP	15:02	15:20
22	late FP	15:46	17:59
23	mid-LP	19:07	15:32
24	late FP	14:19	18:25

*FP: Follicular Phase; LP: Luteal Phase. All scanning sessions were cross-day scans.

Supplementary Table S2. Number of valid trials per condition and participant.

Subject ID	Late Follicular Phase (FP)		Mid-Luteal Phase (LP)	
	Delayed	Immediate	Delayed	Immediate
1	29	11	35	4*
2	11	29	8	31
3	33	7	33	7
4	7	33	9	26
5	18	22	24	16
6	24	16	22	18
7	29	11	33	7
8	29	11	30	10
9	23	17	24	16
10	18	22	19	21
11	10	30	22	18
12	31	9	33	7
13	22	18	23	17
14	32	8	31	9
15	27	13	24	16
16	33	7	26	14
17	38	2*	30	10
18	26	14	26	14
19	6	34	11	29
20	26	14	27	13
21	27	13	27	12
22	28	12	30	10
23	17	23	22	18
24	28	12	23	17

*:*These trials were assigned a weight of 0 in the analysis due to an insufficient number of trials for reliable model estimation.

Supplementary Table S3. Diagnostics for Linear Moderation Models.

Model	Diagnostic Measure	Value
**Primary Moderation Model**		
	Sample Size	48 observations (24 participants)
	Bootstrap Resamples	5,000
	Weighting Scheme	√(Harmonic mean of trial counts)
	Weighted Adjusted R^2	0.362
	Weighted Residual Normality	Shapiro-Wilk W = 0.971, p = .299
**Phase-Specific Moderation: LP**		
	Sample Size	24 observations (24 participants)
	Bootstrap Resamples	5,000
	Weighting Scheme	√(Harmonic mean of trial counts)
	Weighted Adjusted R^2	0.315
	Weighted Residual Normality	Shapiro-Wilk W = 0.957, p = .385
**Phase-Specific Moderation: FP**		
	Sample Size	24 observations (24 participants)
	Bootstrap Resamples	5,000
	Weighting Scheme	√(Harmonic mean of trial counts)
	Weighted Adjusted R^2	0.311
	Weighted Residual Normality	Shapiro-Wilk W = 0.975, p = .781

Note: The primary model tested the moderation effect across all data. Phase-specific models examined the moderation effect separately within the Luteal Phase (LP) and Follicular Phase (FP). Bootstrapping was employed to ensure robust inference.

Supplementary Material S1: Sensitivity Analyses for Time-of-Day Effects

S1.1. Overview

To ensure that our primary findings were not confounded by diurnal variations in hormone levels or neural activity, we conducted a series of comprehensive sensitivity analyses focusing on the time of day of the scanning sessions. All sessions occurred between 13:00 and 19:00. The specific time for each session was recorded and analyzed as described below. All analyses were conducted separately for the late follicular phase (FP) and mid-luteal phase (LP) to account for phase-specific contexts.

S1.2. Analyses and Results

S1.2.1. Analysis of Hormone Levels

We tested whether salivary hormone concentrations (estradiol E2 and progesterone P4) were correlated with the time of day of sample collection within each menstrual cycle phase. Pearson correlation between scanning time and hormone levels, separately for the late FP and the mid-LP. Further, we split participants into "Earlier" and "Later" groups separately for each phase and compared hormone levels.The results are detailed in Table S1.1 below. No significant correlations were found between scanning time and progesterone levels in either the late FP or mid-LP (all $|r| < 0.11$, all $ps > 0.30$). Specially, the "Later" scan group had numerically higher E2 levels than the "Earlier" group in the late FP. However, it is critical to note that this finding does not impact the interpretation of our primary results for two key reasons. Our core analyses were specifically focused on progesterone and its role in modulating the dorsal circuit during the mid-LP. The central behavioral and neural findings reported in the main text are robust to the inclusion of time-of-day as a covariate. Crucially, and as reported in the main text, there was no significant main effect of menstrual cycle phase on estradiol levels ($t(23) = -1.28$, $p = .22$, mean difference = -0.61, 95% CI [-1.61, 0.38], Cohen's $d = 0.26$) in our sample. This suggests that the overall endocrine context of our phase comparison was defined by the expected shift in progesterone, not estradiol. Therefore, while this supplementary finding regarding E2 is noted for full transparency, it does not constitute a confound for our primary conclusions regarding progesterone and menstrual cycle phase.

S1.2.2. Analysis of Functional Connectivity

We tested whether our key neural metric—dlPFC-caudate functional connectivity—was influenced by scanning time within each phase. Pearson correlation between scanning time and the condition-specific functional connectivity estimates (FI, FD, LI, LD), analyzed separately for each phase. No significant correlations were found between scanning time and functional connectivity in any condition (all $|r| < 0.24$, all $ps > 0.26$). To further rule out a non-linear effect of time, we split participants into "Earlier" and "Later" groups separately for each phase and compared our key measures. No significant were found. The results are detailed in Table S1.1 below.

S1.2.3. Analysis of the Key Connectivity Difference Score

We directly tested whether the critical neural contrast underlying our main finding—the functional connectivity difference between delayed and immediate choices—was related to scanning time within each phase. Pearson correlation between scanning time and the connectivity

difference score (Delayed - Immediate), conducted separately for the late FP and the mid-LP. No significant correlation was found in either the late FP (r = .29, p = .17) or the mid-LP (r = -.09, p= .68). To further rule out a non-linear effect of time, we split participants into "Earlier" and "Later" groups separately for each phase and compared our key measures. No significant differences were found between the "Earlier" and "Later" scanning groups for the connectivity difference score in either the late FP. The results are detailed in Table S1.1 below.

Table S1.1: Comprehensive sensitivity analyses for time-of-day effects on hormone functional connectivity

Condition	Correlation with Scan Time		Group Comparison (Earlier vs. Later Scan)		
	r	*p*-value	*t*-value	*p*-value	Cohen's *d*
**E2**					
Late FP	.29	.17	-0.76	.45	0.31
Mid-LP	.34	.11	-1.95	.06	0.80
**P4**					
Late FP	-.07	.74	0.61	.55	0.25
Mid-LP	-.11	.60	0.30	.77	0.12
**Functional Connectivity**					
FI	-.16	.45	0.01	.99	0.004
FD	-.24	.26	1.32	.20	0.54
LI	.17	.43	-0.44	.66	0.18
LD	.12	.58	-0.89	.38	0.36
**Connectivity Difference**					
Late FP	.29	.17	-1.42	.17	0.58
Mid-LP	-.09	.68	0.41	.68	0.17

Note: All analyses revealed no significant effects of time-of-day on functional connectivity measures. The "Earlier" and "Later" groups were defined by a median split of scanning times within each phase for the group comparison. FI: late-FP, immediate; FD: late-FP, delayed; LI: mid-LP, immediate; LD: mid-LP, delayed.

S1.2.4. Time-of-Day as a Covariate in Primary Models

As the most stringent test, we included time-of-day as a continuous covariate in our primary statistical models for behavior and functional connectivity. The covariate was the specific scanning time for the respective phase. The original linear mixed-effects model for functional connectivity and the weighted bootstrap regression for the progesterone moderation analysis were re-run with scanning time included as an additional covariate. In both models, the inclusion of the time-of-day covariate did not alter the significance of our primary findings. The key phase ×

choice type interaction for functional connectivity and the critical three-way interaction for the progesterone moderation analysis remained statistically significant (*ps* < .05).

S1.3. Conclusion

This comprehensive set of sensitivity analyses, conducted separately for each menstrual cycle phase, reveals no significant relationship between the time of day of scanning and our measures of progesterone, functional connectivity, or the critical connectivity difference score. Furthermore, controlling for phase-specific scanning time does not change the significance of our primary results. We therefore conclude that diurnal variation is not a credible confound, and our reported findings robustly reflect the effects of menstrual cycle phase and progesterone.

Furthermore, our supplementary analyses suggested a potential interaction between scan time and menstrual phase for estradiol levels, although the overall phase difference in estradiol was non-significant. This observation highlights an important consideration for future research. The pulsatile and diurnal nature of estradiol secretion may introduce greater variability and pose a challenge for detecting robust cycle-phase effects. Future studies specifically designed to investigate the role of estradiol in cognitive control may benefit from strictly standardizing or explicitly modeling the time of hormone sampling and scanning to better disentangle its effects from diurnal rhythms.

Supplementary Material S2. Motion Sensitivity Analyses

S2.1. Rationale

To quantitatively assess whether our primary functional connectivity finding—the significant phase × choice interaction in dlPFC-caudate connectivity—could be confounded by head motion, we performed two complementary motion-sensitivity analyses.

S2.2. Methods

Head motion was quantified for each participant using the mean framewise displacement (mean FD). The key outcome variable for these analyses was the condition-specific dlPFC-caudate functional connectivity estimate derived from our primary weighted linear mixed-effects model. First, we computed the Pearson correlation coefficient between participants' mean FD and their respective dlPFC-caudate connectivity strength. Then we performed a median split on participants' mean FD to create "Low-Motion" (mean FD ≤ median) and "High-Motion" (mean FD > median) groups. An independent-samples *t*-test was then used to compare the dlPFC-caudate connectivity values between these two groups. A non-significant difference would provide further evidence that the observed connectivity is not spuriously driven by a subset of participants with higher motion.

S2.3. Results

The results of both analyses confirm that our primary finding is robust to the influence of head motion: There was no significant linear relationship between mean FD and dlPFC-caudate connectivity strength across participants (late-FP-delayed: $r = -0.11$, $p = .62$; late-FP-immediate: $r = 0.14$, $p = .53$; mid-LP-delayed: $r = 0.18$, $p = .40$; mid-LP-immediate: $r = 0.19$, $p = .37$). And the dlPFC-caudate connectivity did not differ significantly between the Low-Motion and High-Motion groups (late-FP-delayed: $t(22) = -1.07$, $p = .30$, mean difference = -0.60, 95% CI [-1.75, 0.56], Cohen's $d = 0.44$; late-FP-immediate: $t(22) = -0.12$, $p = .90$, mean difference = -0.05, 95% CI [-0.98, 0.87], Cohen's $d = 0.05$; mid-LP-delayed: $t(22) = 1.70$, $p = .11$, mean difference = 1.03, 95% CI [-0.23, 2.28], Cohen's $d = 0.69$; mid-LP-immediate: $t(22) = 0.50$, $p = .63$, mean difference = 0.15, 95% CI [-0.49, 0.80], Cohen's $d = 0.20$).

S2.4. Conclusion

These supplementary analyses provide quantitative evidence that the reported phase-dependent reversal in dlPFC-caudate connectivity is not a byproduct of head motion during the fMRI scan.
